# Effect of *Bunium persicum* aqueous extract plus endurance exercise on cardiorespiratory capacity and serum lipid profile

**Published:** 2014

**Authors:** Mohammad Khaksari, Mohsen Ahmadi, Hamid Najafipour, Nader Shahrokhi

**Affiliations:** 1 *Physiology Research Center and Department of Physiology, Kerman University of Medical Sciences, Kerman, I. R. Iran *; 2 *Department of Physical Education, Shahid Bahonar University, Kerman, I. R. Iran*

**Keywords:** *Bunium persicum extract*, *Cardiorespiratory capacity*, *Endurance exercise*, *Hypercholesterolemia*, *Lipid profile*

## Abstract

**Objective(s):** We examined the effects of endurance exercise in the presence of *Bunium persicum* extract administration on lipid profile and cardiorespiratory capacity in hypercholesterolemic male mice.

**Materials and Methods** **:** Forty male hypercholesterolemic mice were divided into four groups: Vehicle, Endurance exercise (EE), *Bunium persicum* extract (BPE), and EE + BPE. The exercise protocol was performed at a speed of 18 m/min, 40 min/day, and 5 days/week for 6 weeks. The BPE was administered orally by a dose of 20 mg/Kg/day.

**Results:** The results indicated that the 6-week endurance training accompanied by *Bunium Persicum* extract administration increased cardiorespiratory capacity significantly (601±39 *vs.* 293±20 meters, p<0.001). Total cholesterol level was significantly reduced in EE + BPE compared with Vehicle and EE groups (p<0.05). LDL-c was lower in EE + BPE compared with the Vehicle (p<0.01). HDL-c in BPE and EE + BPE groups was significantly higher than Vehicle (p<0.001 and p<0.05, respectively). Serum triglyceride level was significantly (p<0.05) lower in BPE than the other three groups. Body weight changes were not significantly different between groups.

**Conclusion:** The results suggested that *Bunium persicum* extract is very useful in improvement of lipid profile in hypercholesterolemic animals. Supplementation of the extract to exercise significantly increased the cardiorespiratory capacity.

## Introduction

Reports show that 17 million people die every year from cardiovascular diseases (CVD), of which about 7.6 million are due to coronary heart disease (Rosmand et al., 2008 ▶). An increase in blood lipids, especially cholesterol, plays an important role in aggravating this disease. Hypercholesterolemia is one of the major risk factors that is responsible for about 80% of coronary and cerebrovascular diseases. Contrary to a common belief that cardiovascular disease is a disease of rich men in developed nations, over 80% of CVD deaths take place in low and middle income countries (Asghary et al., 2008 ▶; Mendis et al., 2007). In the Middle East and Eastern Mediterranean countries including Iran, CVDs are considered as common health and social problem which are increasing dramatically. According to the reports from lipid and glucose study in Tehran, 8.8% of adult men and 12.7% of adult women were suffering from cardiovascular diseases in 2002 (Azizi et al., 2002 ▶).

Considering an approximately linear relationship between cholesterol level and mortality due to coronary disease, for each 20 mg/dl increase in total cholesterol level, related mortality rate will increase by 12% (Gaeini and Rajabi, 2005 ▶). Hence, adjusting lipid level is an important factor in health affairs. Undoubtedly, suitable exercise habits play an important role in this adjustment. Most of the researchers believe that physical activity (aerobic type) with moderate intensity, even of little frequency in a week, may lead to a decrease in beta lipoprotein and triglyceride, while an activity with a higher intensity, for at least two months, can result in a decrease in LDL and an increase in HDL (Dustine, 1994 ▶; Hawley, 1998; Rimmer, 1997 ▶; Stein, 2002 ▶).

On the other hand, taking herbal medicines in their traditional way has been issued a great deal in the treatment of a lot of illnesses and an improvement in sport-related abilities (strength and endurance) (Fallah, 2006 ▶; Kessler, 2001 ▶). A lot of researches have been conducted to study the effects of herbal medicine on lipid profile. For instance, Alhasan et al. (2006) reported that margarine herbal supplements and aerobic training resulted in changes in blood enzymes, lipoproteins, and lipids. In this regard, caraway (*carum* *carvi*) can decrease serum triglyceride, cholesterol, and body weight in normal and diabetic rats (Lemhadri, 2006) and prevent oxidative tissue injuries (Dadkhah and Fatemi, 2011 ▶). Therefore, it probably has a similar effect as physical activity on lipid profile. The effects of physical activity are similar to those of *black caraway* on lipid profile and body weight, in addition to an improvement in cardiorespiratory capacity. As there is a significant similarity between *black caraway* (called “Zireh Siah” in folk medicine) and *Bunium persicum *(*B. persicum*) (called “Zireh Koohi”) (Lemhadri et al., 2006), it is anticipated that having physical activity accompanied by *B. persicum* administration amplifies improvement in lipid profile and cardiovascular variables. Therefore, in the present study, we aimed to assess the effect of endurance exercise along with oral administration of aqueous extract of *B. persicum *on lipid profile and cardiorespiratory capacity in a model of hypercholesterolemic animals (male mice).

## Materials and Methods

Experiments were performed on 40 adult male mice, weighing 30-40 g. The animals were housed under standard environmental conditions (231 C, and a 12 h light/12 h dark cycle) with free access to water and standard diet. Study protocol was approved by the Ethic Committee of the Kerman University of Medical Sciences, Iran (Ethic code No. 85/86 KA).

**Induction of hypercholesterolemia**

In order to induce hypercholesterolemia, animals were given a high cholesterol diet including standard chow supplemented with 2% pure cholesterol and 0.5% cholic acid for 4 weeks. Cholic acid was used to dissolve cholesterol before being added to the chow. This was necessary as adding cholesterol without cholic acid did not induce hypercholesterolemia in mice (Luo, 2008 ▶). Blood samples were taken from the tail vein at the beginning and at the end of the period of four weeks to assure induction of hypercholesterolemia.

**Preparation and oral administration of** ***Bunium persicum*** **aqueous extract **

*B. persicum *fruit was purchased from Bazar in Kerman and confirmed by a Pharmacogenosist in Department of Pharmacogenosy, Faculty of Pharmacy, Kerman University of Medical Sciences, Iran (herbarium number kf 1141). Ten grams of powdered fruit of *B. persicum *was mixed with 1000 ml of distilled water, boiled for 10 minutes, and then cooled. Thereafter, the solution was filtered using a Millipore filter to remove particulate material. The filtrate was then freeze-dried (Freeze drier, Eyela, Japan). The dried sample was kept away from moisture in -20 C. For administration, 8 mg of *B. persicum *extract was reconstituted in 4 ml of distilled water (making 2 mg/ml solution) and then given daily using an intergasteric tube (gavage) (1 ml/100 gr BW equal to 20 mg/kg body weight) (Lemhadri, 2006). The extract solution was prepared daily, just before administration. 

**Endurance exercise protocol**

The exercise protocol in two exercised groups was similar to that of Al-Jarrah and colleagues (Al-Jarrah et al., 2007 ▶). A six-lane motorized rodent treadmill (Tecmachine, France) was used for exercise training. The exercise groups of animals were introduced to treadmill slowly over the course of a week with initial orientation and walking on the moving treadmill. The 6-week exercise protocol did not begin until induction of hypercholesterolemia and mice could run at a speed of 18 m/min. The exercise protocol was individualized for each animal, with an aim of running for 40 min/day for 5 days/week at a speed of 18 m/min.

**Experimental design**

After acclimatization for a week, animals were put on hypercholesterolemia regimen for four weeks (see above) and then randomly assigned to four groups of 10 including: Control (vehicle, received distilled water), Endurance exercise (EE), *B. persicum *extract administered (BPE), and EE + BPE groups. All groups underwent their special therapeutic protocol for 6 weeks while hypercholesterolemia regimen was continued during this period. 

The BPE group received the extract for 5 days/week and training groups were put on animal treadmill for endurance exercise. The training in related groups lasted for 6 weeks. The EE + BPE received the *B. persicum *extract 2 hours before exercise each day while EE group received the same volume of distilled water. The control groups received a daily dose of 1 ml/100 gr body weight distilled water during this period and were also put on non-running treadmill (Al-Jarrah, 2007 ▶). This means that the control and BPE groups did not exercise but were transported daily to the training room were exposed to the same environment as the exercised groups of animals.

**Weight measurement**

The animal’s body weight was measured at the beginning and end of experiments (24 hours after the last session of weekly training) using a digital scale with 0.1 gram precision (Grampresicion digital scale, Canada)

**Assessing cardiorespiratory capacity**

Before starting the 6-week training program, all mice underwent a test to assess their capacity for endurance running in five consecutive days. The test was performed between 8 and 12 am. Assessing exercise capacity was according to speed-ramped treadmill to exhaustion (Luo, 2008 ▶). The test was performed as follow: at first, the mouse ran at a speed of 10 m/min and 0 degree slope, then, 2 m/min was added to the speed every two minutes until the mouse became exhausted. Exhaustion was referred to the time that the animal was not able to keep up with the speed of the treadmill for three times and preferred to bear the machine's shock. The machine would go off at the time of exhaustion and the exact time was registered to calculate total running distance (in meters). The best performance during 5 days before and the day after 6-week training program (as pre-test and post-test values, respectively) was considered as cardiorespiratory capacity (Al-Jarrah et al., 2007 ▶).

**Serum lipids and lipoproteins measurement**

Animals' tail blood samples were taken after a 12-hour fasting at the beginning and 6 weeks after initiation of experimental protocol at a similar condition. Serum samples were analyzed enzymatically for the level of total cholesterol (TC), triglyceride (TG), and high density lipoprotein (HDL-c) using commercially available diagnostic kits. Low density lipoprotein was calculated according to the formula: LDL-c = TC- (HDL-c + TG/5). 

**Statistical analysis**

Data in the text and figures are expressed as meanSEM. One way ANOVA was used to compare between groups using the SPSS15 computer package for windows. Post-hoc test was preformed for intergroup comparisons using HSD test. Differences were considered to be significant when p<0.05.

## Results

**Body weight**

There was no significant change in body weight during the course of experiment between groups. Body weight changed in Vehicle from 34±2.5 to 32.8±1.6, in EE from 35.4±1.8 to 33.8±1.9, in BPE from 33.8±1.1 to 31.5±1.5, and in EE + BPE from 32.3±1.4 to 28.9±1.2 grams.

**Cardiorespiratory capacity**

**Figure 1:**
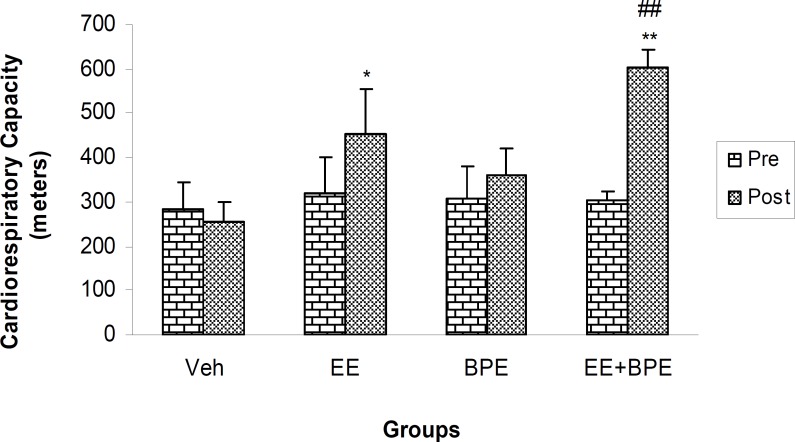
Pre- and post-intervention values for cardiorespiratory capacity in control (Vehicle) and experimental groups. There was no significant difference between the initial (pretest) values in all groups. *=p<0.05, **=p<0.01, compared with related (post-test) control group. ##=p<0.01 compared with endurance exercise (EE) and Bunium persicum extract (BPE) groups

Figure 1 shows the initial and final cardiorespiratory capacity values in control (Vehicle) and experimental groups. No significant difference was found between the initial values in all groups. The difference existed between post-test values of EE with control (p<0.05), EE + BPE with both EE and BPE groups (p<0.05), and EE + BPE with control (p<0.01), with highest increase in capacity in EE + BPE group. 

**Lipid profile**

**Figure 2:**
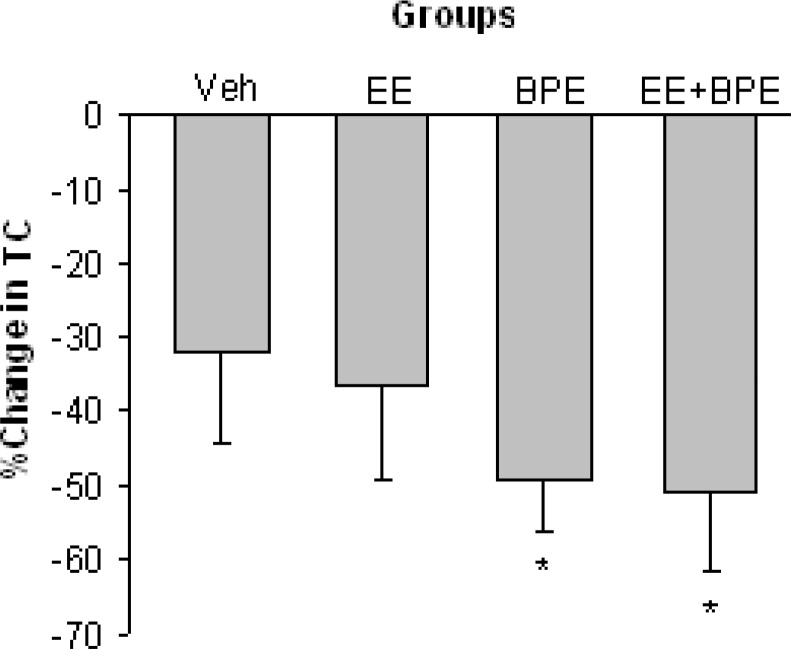
Changes in serum total cholesterol (TC) level in experimental groups. TC was significantly (p<0.05) reduced in BPE and EE + BPE groups compared with Vehicle. *=p<0.05 compared with Vehicle group

Figure 2 shows changes in total cholesterol (TC) level in experimental groups. TC was significantly reduced in BPE and EE + BPE groups compared with control group (p<0.05). The basal (end of hypercholesterolemia induction period) values of TC were 213.1±12.3 in Vehicle, 220±12.5 in EE, 242.1±6.7 in BPE, and 297.7±10.8 mg/dl in EE + BPE groups (The normal value of TC in mice is around 100 mg/dl). The cholesterol value of the EE+BPE group was significantly higher compared with the vehicle group (p<0.05).

**Figure 3:**
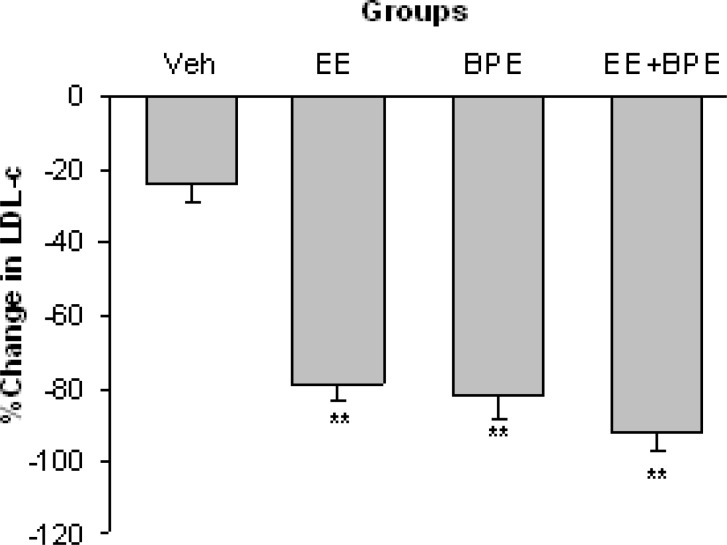
Changes in serum low density lipoprotein cholesterol (LDL-c) level in experimental groups. All three interventions reduced LDL-c significantly. **=p<0.01 compared with vehicle group

The changes in LDL-c are shown in Figure 3. The changes in this variable follow more or less the same trend of changes in TC. LDL-c was also significantly reduced in EE, BPE, and EE + BPE groups compared with control group (p<0.01). The basal (end of hypercholesterolemia induction period) values of LDL-c were 104.1±7.6 in Vehicle, 112.3±4.1 in EE, 143.2±6.7 in BPE, and 151.8± 5.1 mg/dl in EE + BPE groups (The normal value of LDL-c in mice is around 55 mg/dl).

**Figure 4:**
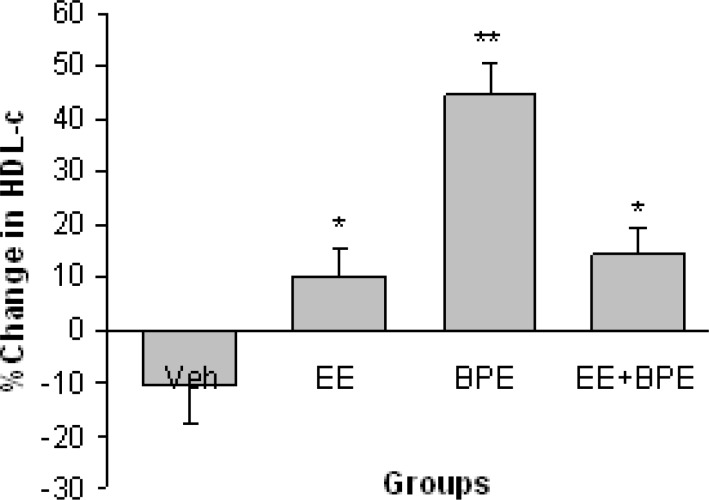
Changes in serum level of high density lipoprotein cholesterol (HDL-c) in experimental groups. All three interventions increased HDL-c significantly but B. persicum extract was more potent than the other two. *=p<0.05, **=p<0.01, compared with vehicle group

Figure 4 shows changes in HDL-c level in experimental groups. All three interventions increased HDL-c significantly (p<0.05 for EE and EE + BPE groups, p<0.01 for BPE group). The basal (end of hypercholesterolemia induction period) values of HDL-c were 92.4 ± 5.6 in Vehicle, 81.5 ± 5.9 in EE, 75.9±6.1 in BPE and 101.4±5.1 mg/dl in EE + BPE groups (The normal value of HDL-c in mice is around 30 mg/dl).

**Figure 5:**
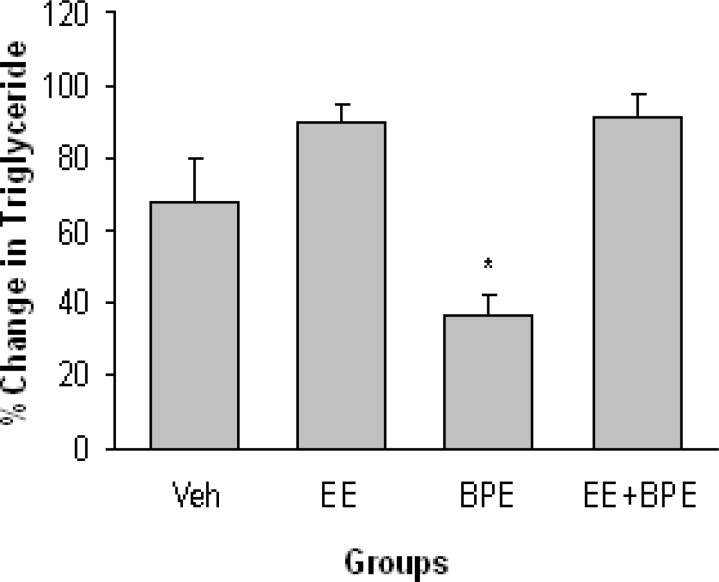
Changes in serum triglyceride (TG) level in experimental groups. TG was increased in all groups, but this increment was significantly less in animals receiving Bunium persicum extract. *=p<0.05 compared with the other three groups

The changes in serum triglyceride (TG) level are shown in Figure 5. TG was increased in all groups, but this increment was significantly (p<0.05) lower in BPE compared with the other three groups. The basal (end of hypercholesterolemia induction period) values of TG were 80.4±12 in Vehicle, 75.8±5.4 in EE, 113.5±5.7 in BPE, and 93.1±6.9 mg/dl in EE + BPE groups (The normal value of TG in mice is around 75 mg/dl).

## Discussion

The present study was designed to investigate the influence of oral administration of aqueous extract of *B. persicum *fruit for 6 weeks, alone and in combination with endurance exercise, on cardiorespiratory capacity and serum lipids, as well as on body weight in hypercholesterolemic mice. 

The results showed a significant increase in cardiorespiratory capacity in the group taking extract together with endurance exercise. Despite improvement in serum lipid conditions, the group taking *B. persicum *alone did not set a significant higher record (Figure 1). This is true for sport nutrition supplements as well, as they cannot improve body ability in the absence of body activity. However, *B. persicum *in combination with exercise potentiatively increased cardiorespiratory capacity (Figure 1) that is a positive finding in regard to cardiovascular health improving strategies. 

The aqueous extract of *B. persicum per se* caused significant decrease in TC, LDL-c, and TG concentrations along with significant increase in HDL-c (Figures 2-5). These show that *B. persicum *may be very effective for improving lipid profile in hyperlipidemia. *B. persicum *was even more effective than exercise, as it had a better effect on increasing HDL-c and on reducing TG along with an equal potency in decrement of LDL-c. Dhandapani and colleagues assessed the effect of green caraway (*Cuminum cyminum L*.) on lipid profile of diabetic rats (Dhandapani et al., 2002 ▶). They gave rats a diet containing green caraway for 6 weeks and concluded that this plant causes significant decrease in TG and TC in diabetic rats. The probable reason for change in lipid profile in the present study is the anti-oxidative effect of *Bunium persicum*. These results could be addressed to *B. persicum *effect considering its structural similarity with green caraway (Haidari and Seyed-Sadjadi, 2011 ▶). Lemhardi and coworkers also evaluated the effect of black caraway extract on two groups of healthy and diabetic rats and reported significant reduction in TC and TG in both groups (Lemhardi et al., 2006). HDL-c and LDL-c were not measured in their study. They suggested four possible mechanisms for lipoprotein reducing effect of black caraway including binding of cholesterol to bile acids in small bowel, increase in bile acids secretion, reduction in activity of 3-hydroxy-3-methylglutaryl coenzyme A reductase (the key enzyme for cholesterol regeneration) and decrement of NADPH needed for cholesterol and fatty acid synthesis (Sharma et al., 2003 ▶).

In another study, Haidari and colleagues reported reduction of TC and LDL-c but no change in TG level due to caraway administration in diabetic rats (Haidari et al., 2011 ▶) (. It has also been reported that black caraway can improve hypercholesterolemia by balancing lipoprotein metabolism which means more LDL-c usage via increase in its receptors and/or increase in lecithin cholesterol acyltransferase (LCAT) (Khanna et al., 2002 ▶). LCAT plays a key role in the combination of free cholesterol with HDL-c and its reverse transfer to VLDL-c or LDL-c in order to return to hepatic cells (Rajlakshmi et al., 2004 ▶). Black Caraway can also facilitate LDL-c catabolism. According to the structural similarity between *B. persicum *and black caraway, these mechanisms probably could also be attributed to the effects of *Bunium persicum*. Although the basal (pre-intervention) value of TC was higher in BPE and BPE + EE groups (Result section), the maximum reduction in TC also belonged to these groups (more than 50%, Figure 2) confirming the effectiveness of *B. persicum *extract as a good cholesterol-lowering agent. If this finding is reproduced in clinical studies, it may be recommended as a therapeutic strategy for cholesterol lowering in hypercholesterolemic patients.

Exercise group results are consistent with the results of similar studies conducted on animals (Ensign et al., 2002; Venditti and Dimeo, 1996 ▶). These are also similar to the finding of a study conducted by Ravikiran and coworkers in which they showed a decrease in the level of TC, TG, LDL-c, and an increase in HDL-c in rats having a 4 week swimming training (6 days a week), with the exception that in the present study, TG increased in all groups (Ravikiran et al,. 2006 ▶). In that study, a 40-minute practice a day (the same as training duration in our study) led to decrease in TG. The reason that TG level in the present study was changed reverse to the anticipated direction is not known at present but it may be due to the colic acid added to the regimen (method section above) to induce hypercholesterolemia in mice as cholic acid is one of the components of the bile that facilitates fat digestion and absorption. In this regard, higher increases in TG in the exercised groups (EE and EE + BPE) compared with the other two groups (Figure 5) may be due to the higher food consumption (more cholic acid ingestion) in these groups to compensate their higher energy expenditure. 

Results from another part of the study have shown that endurance training and *B. persicum *did not change body weight. Our results were in contrast to the results of Asha Devi and colleagues in which mice's weight increased after 2 months of training (Asha Devi et al., 2003) and in contrast with the findings of Lemhadri and colleagues in which they showed significant body weight loss (Lemhadri et al., 2006). In addition, these results are different from those of Dhandapani and colleagues in which they reported weight gain after using green caraway (Dhandapani et al., 2002 ▶). Intensity of training program in the present study has helped to keep animals' body weight stable during training period in some ways. Not changing body weight could be an advantage for *Bunium persicum*, as overweighting and obesity are predisposing factors for coronary artery diseases. 

Overall, the results of the present study show that *B. persicum *extract have a very beneficial effect on lipid profile. When its administration is accompanied by endurance training leads to significant increase in cardiorespiratory capacity. These along with keeping body weight stable which are all beneficial for prevention of cardiovascular diseases, candidate this plant for conducting more researches about its potential therapeutic application. At present, complementary studies are needed to determine the exact mechanism of *B. persicum *actions on cardiorespiratory capacity. 
